# Understanding the Fate of Almond (*Prunus dulcis* (Mill.) D.A. Webb) Oleosomes during Simulated Digestion

**DOI:** 10.3390/nu12113397

**Published:** 2020-11-05

**Authors:** Domenico Trombetta, Antonella Smeriglio, Marcella Denaro, Roberto Zagami, Mara Tomassetti, Rosa Pilolli, Elisabetta De Angelis, Linda Monaci, Giuseppina Mandalari

**Affiliations:** 1 Department of Chemical, Biological, Pharmaceutical and Environmental Science, University of Messina, Via Giovanni Palatucci, 98168 Messina, Italy; dtrombetta@unime.it (D.T.); asmeriglio@unime.it (A.S.); mdenaro@unime.it (M.D.); 2 CNR-ISMN c/o Department of Chemical, Biological, Pharmaceutical and Environmental Sciences of the University of Messina, V.le F. Stagno d’Alcontres 31, 98166 Messina, Italy; roberto.zagami@ismn.cnr.it; 3 Dompé Farmaceutici S.p.A., Via Pietro Castellino, 80131 Napoli, Italy; mara.tomassetti@dompe.com; 4 Institute of Sciences of Food Production, National Research Council of Italy (ISPA-CNR), via Giovanni Amendola 122/O, 70126 Bari, Italy; rosa.pilolli@ispa.cnr.it (R.P.); elisabetta.deangelis@ispa.cnr.it (E.D.A.); linda.monaci@ispa.cnr.it (L.M.)

**Keywords:** almonds, in vitro digestion, bioaccessibility, oleosomes

## Abstract

Background: Almond kernels contain phytochemicals with positive health effects in relation to heart disease, diabetes and obesity. Several studies have previously highlighted that almond cell wall encapsulation during digestion and particle size are factors associated with these benefits. In the present study, we have characterized almond oleosomes, natural oil droplets abundant in plants, and we have investigated their integrity during simulated gastrointestinal digestion. Methods: Oleosomes were visualized on the almond seed surface by imaging mass spectrometry analysis, and then characterized in terms of droplet size distribution by dynamic light scattering and protein profile by liquid chromatography high-resolution tandem mass spectrometry analysis. Results: The almond oleosomes’ distribution remained monomodal after in vitro mastication, whereas gastric and duodenal digestion led to a bimodal distribution, albeit characterized mainly by a prevalent population with a droplet size decrease related to a rearrangement of the protein profile. Oleosins, structural proteins found in plant oil bodies, persisted unchanged during simulated mastication, with the appearance of new prunin isoforms after gastric and duodenal digestion. Conclusions: The rearrangement of the protein profile could limit lipid bioaccessibility. The data improve our understanding of the behavior of almond lipids during gastrointestinal digestion, and may have implications for energy intake and satiety imparted by almonds.

## 1. Introduction

Almond lipids, representing about 55% of nut weight, are stored as triacylglycerols (TAG) in oil bodies called oleosomes, which are lipid droplets of a diameter typically between 0.2 and 2.0 μm, surrounded by a monolayer of phospholipids embedding proteins [1,2]. These proteins are mainly oleosins with low molecular weight (16–24 kDa). One protein domain covers the phospholipid surface, a second penetrates the monolayer, and a third anchors to the triglyceride matrix. These features prevent lipid hydrolysis by phospholipases and stabilize oil bodies from coalescence [3]. In addition to a stabilizing role, oleosins are possible receptors for lipase binding during seed germination, and consequently may play a key role in interacting with human gastrointestinal enzymes.

In a previous study, we showed that lipids are evenly distributed in natural raw almonds and are located in oleosomes surrounding the protein bodies [4]. Furthermore, individual undamaged cells containing lipids remaining within oleosomes were identified in chewed whole raw samples [5]. We have also shown that in fecal samples of volunteers fed with whole raw almonds [6], the lipid contents of the cells at the periphery of the almond particles were in the form of large drops. However, it was not possible to observe whether the lipid in the innermost cells of the larger particles were still within the oleosomes [5].

A number of studies have highlighted the importance of almond cell wall encapsulation in the kinetics of lipid and other macronutrients’ digestion, with consequences for reduced energy absorption and postprandial lipaemia [7,8,9,10]. These mechanisms could contribute to a better understanding of the benefits related to almond consumption for human health [11].

Some evidence of oleosomes in almond tissue subjected to in vitro digestion has previously been described [4]. However, the distribution of oleosomes within the almond seeds, as well as their structural stability following simulated human digestion, have not been investigated.

Considering this, the aim of the present work was to investigate the nature of almond oleosomes and their integrity maintained during passage through the gastrointestinal (GI) tract using a simulated human digestion model. We also provide images of the distribution of almond oleosomes within the seed with their sophisticated monolayer membrane of phospholipids and anchored unique proteins. The determination of the lipid profile in seed extracts gives detailed information about amounts and types of lipid. However, lipid localization often remains unknown due to losses during sample preparation and extraction. Matrix-assisted laser desorption/ionization (MALDI) imaging mass spectrometry (MS) represents the best method for a label free visualization of the most representative lipids present in oleosomes. Lipids are particularly well-suited for this technique since they ionize in a predictable manner, and extensive databases are currently available for their identification [12].

## 2. Materials and Methods

### 2.1. Imaging Mass Spectrometry Analysis

Natural raw unsalted almonds were kindly provided by the Almond Board of California. 

A cryostat almond section (12 μm) was deposited on glass, coated with Indium Tin Oxide (ITO glasses), and covered with the matrices 2,5-dihydroxybenzoic acid (DHB) and 9-aminoacridine (9-AA) before analysis. Specifically, for matrix assisted laser desorption/ionization imaging mass spectrometry (MALDI-IMS) in the positive ion mode, 50 mg/mL of DHB in methanol/water (7/3, *v/v*) was sprayed uniformly over the sample section using an automatic pneumatic ultrafine sprayer (“SMALDIPrep”, TransMIT GmbH, Giessen, Germany) [13]. For MALDI-IMS in the negative ion mode, 9-AA was prepared at a concentration of 10 mg/mL in ethanol/water (7/3, *v/v*), and used as the matrix. IMS experiments were performed using the high-resolution atmospheric-pressure scanning microprobe matrix assisted laser desorption/ionization ion source AP-SMALDI-10 (TransMIT GmbH, Giessen, Germany) coupled to an orbital trapping ion mass spectrometer Q Exactive (Thermo Scientific GmbH, Bremen, Germany). A nitrogen gas laser (LTB Lasertechnik GmbH, Berlin, Germany) with a wavelength of 337 nm, operating at a repetition rate of 60 Hz, was used. The laser beam was focused coaxially with the ion beam by a centrally bored objective lens [14]. The mass spectrometry parameters were set to obtain the highest sensitivity with m/z values in the range of 400–1000 m/z in the positive ion mode and 300–1000 m/z in the negative ion mode. Mirion software (TransMIT GmbH, Giessen, Germany) was used for the automatic acquisition of the spectra, the reconstruction of the ion images, and for the total ion current normalization. The lipid species were annotated based on accurate ion mass (m/z) values, by using databases such as LIPID MAPS (www.lipidmaps.org), METLIN (www.metlin.scripps.edu) and the Human Metabolite Database (www.hmdb.ca). In the negative ion mode, deprotonated [M − H]^−^, and in the positive ion mode protonated [M + H]^+^, sodiated [M + Na]^+^ and potassiated [M + K]^+^ species, were considered for the annotation.

### 2.2. Extraction of Almond Oleosomes

Oleosome extraction and purification was carried out according to De Chirico et al. [15] with some modifications in three independent experiments (*n* = 3). Whole almonds (~1 g) were soaked in sodium bicarbonate (0.1 M, pH 9.5, 1:4 *w/w*) containing 0.02 mM sodium azide (in order to avoid microbial spoilage) at 4 °C for 16 h. The soaking medium was then discarded and the sample was ground in the same pre-cooled (4 °C) medium (1:7, *w/w*) with an IKA ULTRA-TURRAX^®^ T45 homogenizer (IKA^®^-Werke GmbH & Co. KG, Staufen, Germany).

The obtained slurry was left stirring for 24 h at 4 °C, in order to improve the yield of oleosomes, and then centrifuged at 10,000× *g* for 30 min at 4 °C. The upper layer was isolated using a spatula and drained on filter paper. The crude oleosomes fraction was subjected to a partial purification step by dispersion into a washing solution (0.1 M NaHCO3, pH 9.5, 1:4 *w/w*) followed by centrifugation (10,000× *g*, 30 min at 4 °C). In order to remove any residue of the washing solution, the fat fraction was isolated using a spatula, drained on filter paper, suspended in water (1:4 *w/w*) and centrifuged (10,000× *g*, 30 min at 4 °C).

The cream layer was then collected in a burnished glass vial with nitrogen headspace and stored at 4 °C until subsequent analyses. The average extraction yield was 34.59% ± 0.765.

### 2.3. Characterization of the Protein Fraction Embedding in Phospholipid Layer

On three different aliquots of pure almond oleosomes, an efficient extraction protocol was optimized and applied aiming at disaggregating the oleosome micelle and increasing the protein yield in the final extract. More specifically, the suitability of two different buffers was tested for the effective extraction of proteins from the oleosome and the best conditions were found by comparing a solution of 2% w/v Sodium Dodecyl Sulphate (SDS) in water with 10 mM sodium phosphate buffer at pH 7.5. Proteins were extracted according to the following protocol: approximately 200 mg of cream layer containing almond oleosomes was combined with 4 mL of extraction buffer, mixed for 3 min and subjected to ultrasonic treatment with a probe sonicator (Vibracell^TM^, Sonics & Materials Inc., Newton, CT, USA) under the conditions of time of 20 min, amplitude of 70, and pulse lengths of 10 s ON/2 s OFF (overheating was controlled with an ice bath). The extract was then stored at room temperature until use. The extraction capacity of proteins was assessed by the commercial bicinchoninic acid (BCA) protein assay kit (G-Biosciences, St. Louis, MO, USA), according to the manufacturer’s instruction, in order to select the procedure capable of providing the best extraction yield. The obtained protein extracts were then separated by sodium dodecylsulphate-polyacrylamide gel electrophoresis (SDS-PAGE) on 8–16% polyacrylamide pre-cast gels (8.6 cm × 6.7 cm × 1 mm, Bio-Rad Laboratories, Segrate, MI, Italy). Before gel analysis, the samples were mixed (1:1 volume ratio) with a Laemmli buffer (62.5 mM Tris-HCl, pH 6.8, 25% glycerol, 2% SDS, 0.01% bromophenol blue, 100 mM dithiothreitol) and then kept at 95 °C for 5 min to allow protein denaturation. Specifically, an equal volume of protein extracts was loaded on each lane (30 µL corresponding to approximately 50 µg of proteins). PAGE gels were run in the Mini-Protean Tetra Cell equipment (Bio-Rad Laboratories, Segrate, MI, Italy) with Tris-glycine-SDS (TGS) running buffer (25 mM Trizma Base, 192 mM glycine, 0.1% SDS) under the following conditions: 60 V for 10 min and 90 V until the end of the run. Gels were stained using a solution of Coomassie brilliant blue G-250 for approximately 90 min, and the bands were detected on a ChemiDoc imaging System (Bio-Rad Laboratories, Segrate, MI, Italy). For protein molecular weight referencing, 5 µL of a Precision Plus Protein™ All blue standard (10–250 kDa, Bio-Rad Laboratories, Segrate, MI, Italy) was loaded on each gel. Concerning the oleosome derived from the chewing and gastric and duodenal digestion of almonds (please refer to Section 2.7 for experimental conditions), the proteins were firstly extracted by following the procedure above described, employing a solution of 2% Sodium Dodecyl Sulphate (SDS) as the extraction buffer. Afterwards, in order to remove interfering compounds (such as ionic contaminants, i.e., detergent) which could affect electrophoresis separation, these protein extracts were submitted to a further clean-up step based on selective precipitation. In particular, 130 µL of extract (corresponding to approximately 5.2 mg of oleosomes) of chewing, gastric and duodenal fluids were purified using the commercial ReadyPrep^TM^ 2-D cleanup kit (Bio-Rad Laboratories, Segrate, MI, Italy) following the manufacturing protocol slightly modified for the washing step. Specifically, in the current procedure, washing solution 1 was retained throughout the steps, contrarily to what is suggested by the kit producer, in order to avoid sample loss. The final protein pellets were dissolved in 30 µL of Laemmli buffer (62.5 mM Tris-HCl, pH 6.8, 25% glycerol, 2% SDS, 0.01% Bromophenol Blue, 100 mM DTT) previously diluted to 1:1 (vol:vol) with water. At these conditions, the final protein content of each sample was concentrated by 4 times. Finally, the purified proteins derived from the chewed, gastric and duodenal samples (final volume of 30 µL) were separated by SDS-PAGE after denaturation (95 °C, 5 min), according to what is described for undigested oleosome investigation.

### 2.4. In-Gel Tryptic Digestion

The most relevant protein bands, detected for the electrophoretic gel of oleosome samples before and after gastroduodenal digestion experiments, were excised from the gel and digested according to the protocol described by De Angelis et al. [16], with few modifications regarding the proteolytic enzyme selected. Specifically, trypsin was replaced by chymotrypsin, thus an appropriate buffer (Tris-HCl 100 mM, pH 8.0 + 10 mM CaCl_2_) was used to prepare dithiothreitol (10 mM stock solution) and iodoacetamide (55 mM stock solution) solutions. Moreover, a calculated amount of sequencing grade chymotrypsin (Promega Corporation, WI, USA. Source: bovine pancreas, activity: 70 units/mg by BTEE) was added to the sample, so that a ratio 1/20 enzyme/protein was attained to facilitate a complete enzymatic digestion. The digestion process was performed at 25 °C, while keeping samples shaking at 500 rpm with an Innova 4000 Benchtop Incubator Shaker (New Brunswick Scientific, NJ, USA), and it finally lasted 16 h. After drying, each digested sample was suspended in 80 µL of H_2_O/ACN 95/5 + 0.1% formic acid (*v/v*) and analyzed by mass spectrometry.

### 2.5. Liquid Chromatography High-Resolution Tandem Mass Spectrometry (LC-HR-MS/MS) Analysis and Software-Based Identification

Untargeted HR-MS/MS analysis was performed on a Q-Exactive™ Plus Hybrid Quadrupole-Orbitrap™ Mass Spectrometer coupled with an Ultra-High-Performance Liquid Chromatography (UHPLC) pump system (Thermo Fisher Scientific, San José, CA, USA). The peptide mixtures obtained from the protein bands in the digested gel referring to raw and GI-digested almond oleosome were separated on a Aeris peptide XB C18 analytical column (internal diameter 2.1 mm, length 150 mm, particle size 3.6 micron, porosity 100 Å, Phenomenex, Torrance, CA, USA) at a flow rate of 200 µL/mL using a direct injection configuration. In particular, all the HPLC and MS instrumental set-ups were described previously [17,18], and the injection volume was set to 20 µL. Raw data were processed by Proteome Discoverer v.2.1 sp1 (Thermo Fisher Scientific, San José, CA, USA) for peptide/protein identification. The Sequest^HT^ searching algorithm was applied against a customized database including the *Prunus dulcis* reference proteome (ID UP000327085, download on 1 October 2020), along with the sequences of all the enzymes used to simulate the human GI digestion. Chymotrypsin was selected as the cleavage enzyme in the analysis of oleosomes extracted from raw almond, whereas ‘no-enzyme’ was selected for GI digesta. In all cases, the mass tolerance on the precursor and fragment ions was set to 5 ppm and 0.05 Da, respectively. As for the processing workflow the fixed value of peptide spectrum match (PSM) validator node was included, setting a maximum delta Cn of 0.05 to be applied to all available PSM. In the consensus workflow, we set 0.01 and 0.05 as the strict and relaxed FDR, respectively, in both the peptide (PSM and peptide) and protein validation nodes. The list of peptides with at least medium confidence (False Discovery Rate—FDR <5%) was screened by the operator checking the peptide spectrum matches, according to the following acceptance criteria: (i) the intensity of the MS/MS spectrum is sufficiently high (higher than 1E4), (ii) the main peaks of the spectrum are assigned to the specific transitions, (iii) at least three peaks are correctly assigned to peptide transitions, preferably y- and b-transitions (with the following restrictions: the transitions are reported in multiple charge states, for example y_n_^+^ and y_n_^++^ are counted only once; the peak of the precursor ion with water loss is not considered as a useful fragment for assignment), (iv) the peptide sequences with proline residues should present preferential fragmentation at the P level (the relevant y-type transition should dominate the MS/MS fragmentation spectrum).

These criteria were already successfully used in previous publications from our Research Group [19,20]. Moreover, as for protein identification, a minimum of two peptides with at least one unique sequence for that accession was set as the threshold for the final assignment.

### 2.6. Droplet Size Distribution

The size distribution and average diameter of undigested and digested oleosomes were determined by dynamic light scattering on a Malvern 4700 submicron analyzer (Malvern Instruments Inc., Worcestershire, UK), equipped with an He−Ne laser at a power P = 4.0 mW and λ = 633 nm. The measurements were performed at a 173° angle with respect to the incident beam at 25 ± 1 °C for each dispersion, utilizing a noninvasive back-scattering (NIBS) technique. Distilled water was used as the dispersant. The cumulative mean diameter (z-average) and polydispersity index (PdI) were used to describe the droplet average size and size distribution, respectively.

PdI values close to 1.0 are indicative of polydisperse systems [21], whereas values close to 0.0 suggest monomodal systems.

### 2.7. Simulated Human Digestion

#### 2.7.1. Chemicals and Enzymes

Sodium chloride (NaCl), potassium chloride (KCl), calcium chloride (CaCl_2_), urea, cholesterol, sodium phosphate monobasic (NaH_2_PO_4_), zinc sulphate heptahydrate (ZnSO_4_·7H_2_O), α-amylase from human saliva type XI (A1031-1KU), egg-phosphatidylcholine (PC, 840051P), pepsin from porcine gastric mucosa (P6887), α-chymotrypsin type II from bovine pancreas (C4129), trypsin type IX-S from porcine pancreas (T0303), lipase type VI-S from porcine pancreas (L0382), colipase from porcine pancreas (C3028), α-amylase type VI-B from porcine pancreas, sodium glycodeoxycholate (G9910) and taurocholic acid sodium salt hydrate (T4009) were purchased from Merck KGaA (Darmstadt, Germany). The lipase for the gastric phase of digestion was a gastric lipase analogue of fungal origin (F-AP15) from Amano Enzyme Inc. (Nagoya, Japan).

#### 2.7.2. Chewing

The aim of this procedure was to simulate the chewing of the almond meals in the mouth. Chewing is the initial step in the digestion process and this procedure was designed to simulate both the salivary amylase activity and the mechanical breakdown of the food. Natural almonds (25 g) were minced three times using a mincer (Edité par totally Addict^®^ Groupe CMP 157, Le Blanc-Mesnil, France) in order to simulate the mechanical oral breakdown of the meal. Thereafter, 900 U of Human Salivary Amylase (HSA) was dissolved in 1 mL Simulated Salivary Fluid (SSF) at pH 6.9 (0.15 M sodium chloride, 3 mM urea), brought to a volume of 12.5 mL, added to the minced almonds, and left in contact for 30 s at room temperature.

#### 2.7.3. Gastric Digestion

The chewed samples (5 g) were subjected to in vitro gastric digestion in 50 mL glass bottles covered with aluminum foil under the following conditions: NaCl (58 mM), KCl (30 mM), CaCl_2_ (0.5 mM), NaH_2_PO_4_ (0.864 mM), egg-phosphatidylcholine (0.127 mM). The pH was adjusted to 2.5 by the addition of 1M HCl, and porcine gastric mucosa pepsin and a gastric lipase were then dissolved at a final concentration of 9000 U/mL and 60 U/mL, respectively.

Samples were incubated for 2h at 37 °C under constant agitation (170 rpm) by an Innova 4000 Benchtop Incubator Shaker (New Brunswick Scientific, NJ, USA).

#### 2.7.4. Duodenal Digestion

After adjusting the pH to 7.5 with 1M NaOH, the gastric samples (2.5 g) were transferred into a 50 mL glass bottle covered with aluminum foil for duodenal digestion with the addition of 4.33 mL of simulated bile solution (12.5 mM sodium taurocholate, 12.5 mM sodium glycodeoxycholate, 6.5 mM dried lecithin and 4 mM cholesterol) and pancreatic enzyme solution (12.17 mL), and incubated at 37 °C under shaking conditions (170 rpm) by an Innova 4000 Benchtop Incubator Shaker (New Brunswick Scientific, NJ, USA) for 4 h. The pancreatic enzyme solution consisted of NaCl (125.0 mM), CaCl_2_ (0.6 mM), MgCl_2_ (0.3 mM), ZnSO_4_·7H_2_O (4.1 μM), porcine pancreatic lipase (590 U/mL), porcine colipase (3.2/mL), porcine trypsin (11 U/mL), bovine α-chymotrypsin (24 U/mL), and porcine α-amylase (300 U/mL).

All digested samples were immediately frozen at −20 °C for the subsequent extraction and characterization of almond oleosomes.

### 2.8. Post Digestion Analyses

The aliquots taken from chewed, post in vitro gastric and duodenal digestion were used to extract oleosomes, using the protocol reported in Section 2.2. The extracted oleosomes were characterized in terms of protein composition and droplet size distribution following the methods reported above.

## 3. Results

### 3.1. Oleosomes Distribution within Almond Seeds

Oleosomes are natural oil droplets (0.2–2.0 μm) with a sophisticated membrane consisting of a monolayer of phospholipids and anchored unique proteins [2]. The determination of the lipid profile in the seed extracts gave detailed information about the amount and types of lipid. However, their localization often remains challenging given the several drawbacks encountered during sample preparation, which can alter the native structure of the seed matrix. For these reasons, MALDI imaging MS proved to be a good approach for a label free visualization of the most representative lipid present in the oleosomes, such as phosphatidylcholine and phosphatidylinositol, and consequently can be useful in identifying their position within the almond.

**Figure 1:**
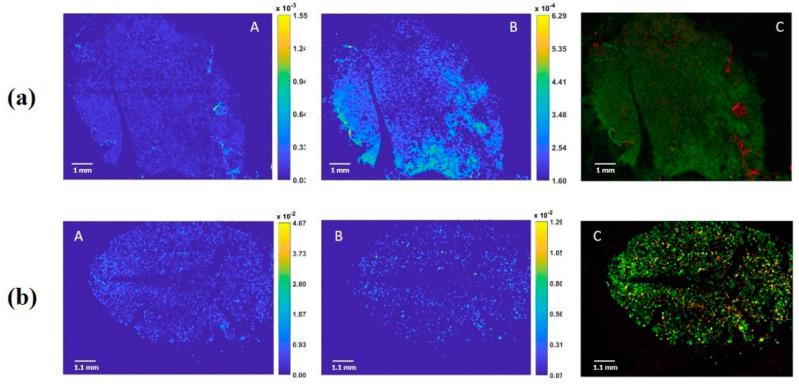
(**a**) Positive ion images (60 μm lateral resolution) of an almond section; A: Phosphatidylcholine 784.5750 [PC(18:1/14:0)+H]^+^; B: Phosphatidylcholine 786.5850 [PC(18:1/18:1)+H]^+^; C: overlay of two lipid signals; (**b**) Negative ion images (60 μm lateral resolution) of almond section; A: Phosphatidylinositol 835.5050 [PI(16:0/18:1)-H]^−^; B: Phosphatidylinositol 861.5450 [PI(18:1/18:1)-H]^−^ or [PI(18:0/18:2)-H]^−^; C overlay of two lipid signals.

Following the signal of the most abundant phospholipids previously identified in Californian almonds (*P. dulcis* L. cv Nonpareil) [20], such as phosphatidylcholine [PC(18:1/14:0)+H]^+^ (m/z: 784.5750) and [PC(18:1/18:1)+H]^+^ (m/z: 786.5850) for the positive ion, we aimed to localize the oleosome alongside the almond sections. We observed that PC (18:1/14:0) was evenly distributed throughout the seed, while the [PC (18:1/18:1)] was localized in certain specific region of the sections (Figure 1a). In contrast, we observed, in the negative ion mode, the distribution of two phosphatidylinositols, such as [PI(16:0/18:1)-H]^−^ (m/z: 835.5050) and [PI(18:1/18:1)-H]^−^ or [PI(18:0/18:2)-H]^−^ (m/z: 861.5450), which showed a homogeneous distribution within the seed (Figure 1b).

### 3.2. Protein Profile

**Figure 2:**
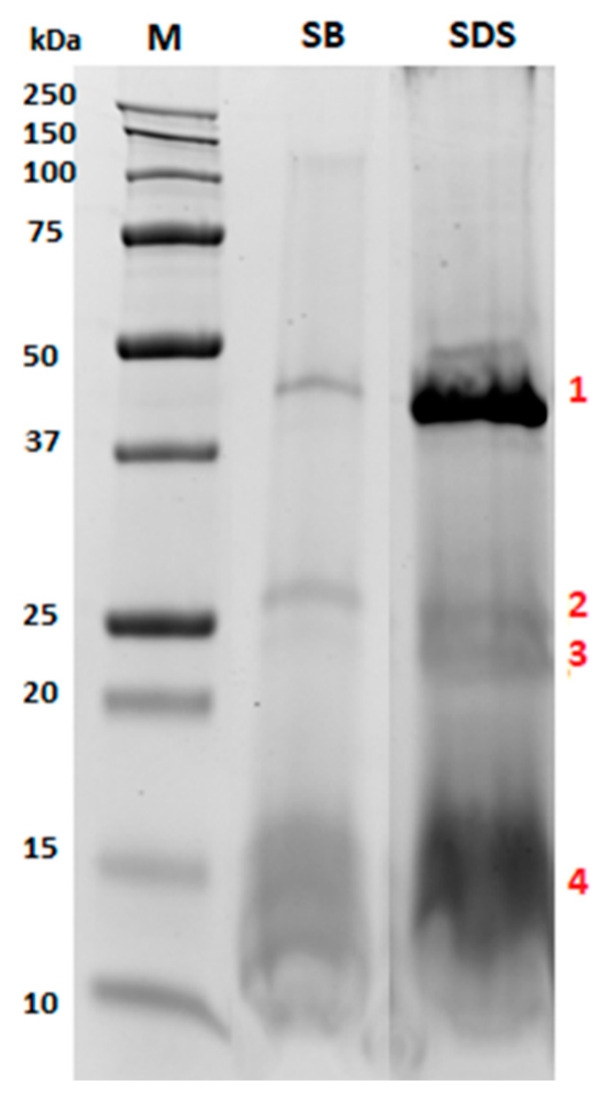
Comparison of the sodium dodecylsulphate-polyacrylamide gel electrophoresis (SDS-PAGE) protein profiles obtained by extracting almond oleosomes either with water 10 mM sodium phosphate pH 7.5 buffer (lane named SB) or 2% SDS (lane named SDS). Numbers in red (1–4) indicate bands that were submitted to in-gel digestion and LC-MS/MS analysis for identification purposes.

Proteins were extracted from almonds by applying the two different extraction protocols herein optimized and tested. The electrophoretic profiles of proteins extracted from undigested oleosomes using the two buffers are illustrated in Figure 2.

As shown in Figure 2, the almond extract pattern did not significantly change between the two selected buffers (sodium phosphate buffer and 2% SDS), meaning that both buffers enabled a satisfactory extraction of the same protein pool, although the intensity of the bands was remarkably different. Specifically, different signals were observed, especially in the ranges of 37–50 kDa, 20–30 kDa and 10–17 kDa. Interestingly, the protein bands indicating the 2% SDS electrophoretic profile appeared more intense, highlighting that the composition of this buffer likely improved the disruption of the phospholipid layer, with consequent solubilization of the embedded proteins. For this reason, this condition was selected for further experiments.

In order to deepen our knowledge on oleosomes protein composition, liquid chromatography high resolution tandem mass spectrometry (LC-HR-MS/MS) experiments were carried out in order to identify the specific proteins of interest. To this end, some selected protein bands from the 2% SDS electrophoretic pattern (Figure 2, lane SDS, bands 1–4) were in-gel digested by chymotrypsin, and the resulting peptide pool was separated using micro-HPLC and subjected to MS/MS analysis according to the experimental conditions detailed in our previous work [16,17,18], with some modification for the in-gel digestion protocol (see Methods section for details). Protein identification was achieved via a commercial software using the Sequest HT searching algorithm against a customized database containing the reference proteome of *Prunus dulcis*. The results are summarized in Table 1.

As expected, the proteins banded in the region of 10–15 kDa (corresponding to band 4) were attributed to two almond oleosins (MW approximately 16 kDa), likely two isoforms of the same protein accessed with the ID A0A5E4ET55 and A0A5E4EAT1. Indeed, oleosins are transmembrane proteins embedded in the lipid layer of oleosomes, which may play a structural role in stabilizing the lipid body during seed desiccation by preventing the coalescence of the oil. Band 1 was attributed to a basic 7S globulin with endopeptidases activity, which belongs to the peptidase A1 family, while bands 2 and 3 to proteins with general cellular functions. The peroxygenase (A0A5E4EKE0) belongs to the caleosin family, which is also expected to be present in oleosomes having an oleosin-like association with lipid bodies in mature seeds associated almost exclusively with storage lipid bodies. The beta-tonoplast intrinsic protein detected in bands 2 and 3 is a transmembrane protein directly involved in the transmembrane transport activity undertaken by energy-independent facilitated diffusion. Interestingly, in bands 3 and 4d, a low amount of a legumin protein was also detected (few peptides identified) assigned to the acid and basic chains of Prunin 1 (accession number A0A5E4FFS0) released under the reducing conditions of the SDS PAGE.

**Figure 3:**
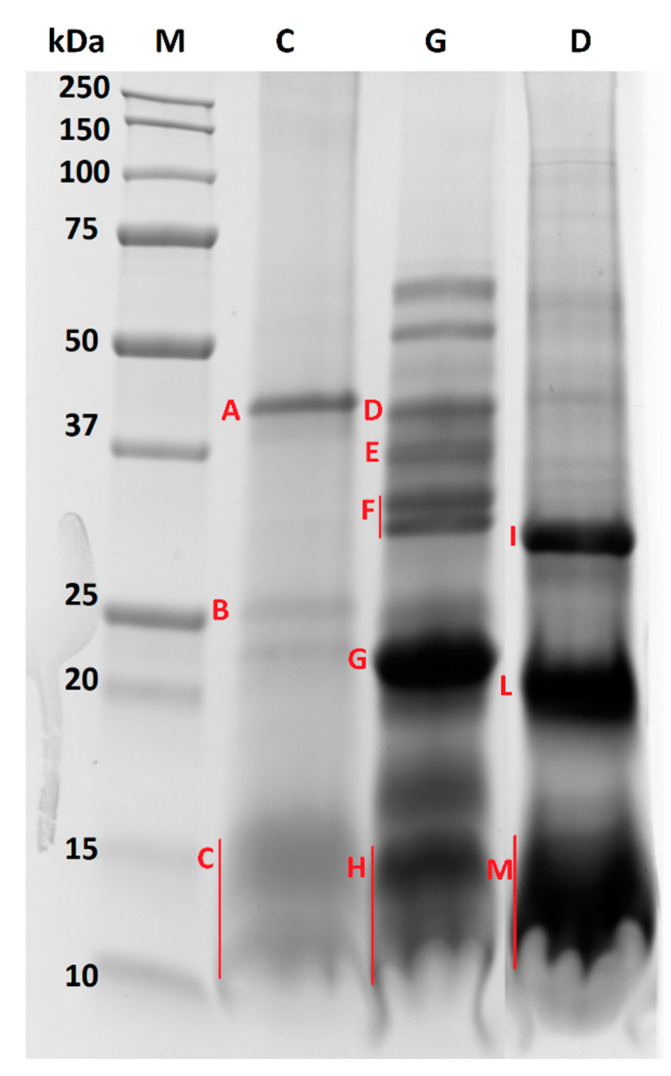
Comparison of SDS-PAGE protein profiles referred to almond oleosomes submitted to chewing (lane C), gastric (lane G) and duodenal (lane D) digestion. Bands submitted to in-gel digestion for protein identification by LC-MS/MS analysis are marked with red capital letters from A to M for GI digested oleosome.

The proteomic analysis was then extended to the oleosome extracted from almond samples submitted to chewing, and gastric and duodenal digestion. The resulting electrophoretic profiles are illustrated in Figure 3.

Interestingly, by visual inspection of the protein profiles of undigested and digested almond oleosomes, we can clearly infer that the stability of the oleosome proteins appeared to change when passing from chewing through to the gastric and duodenal processes. When focusing on the chewed sample (Figure 3, lane C), no differences could be observed compared with undigested oleosomes (Figure 2, lane SDS). Indeed, both samples showed the same bands in the ranges of 37–50 kDa, 20–25 kDa and 10–15 kDa, suggesting that salivary enzymes did not affect the protein profile. On the contrary, the protein profiles obtained after simulated gastric and duodenal digestion (Figure 3, lane G and D, respectively) appeared to change with respect to the chewed sample. Some of the main bands (37–50 kDa, 20–25 kDa and approximately 10 kDa) clearly visible in the chewed sample (Figure 3, lane C) disappeared after gastric digestion (Figure 3, lane G), and new proteins, mainly banding in the range of 50–75 kDa (two distinct bands), 31–42 kDa (four distinct bands) and below 23 kDa, were displayed in the gel. On the contrary, the protein banding at approximately 15 kDa in the chewed sample still persisted after gastric digestion, suggesting a likely resistance to pepsin activity. A different protein profile was observed after duodenal digestion (Figure 3, lane D), which is mainly characterized by the three intense bands visible in the range of 25–37 kDa, and approximately at 20 and 10–15 kDa. Compared to the gastric sample (Figure 3, lane G), the four bands visible in the range of 31–42 kDa disappeared after duodenal digestion, and in their place a new intense band at approximately 30 kDa was visible. A similar behavior was observed for the bands at 15 and 17 kDa of the gastric sample, which appeared to be fully digested after duodenal digestion and no longer visible at the corresponding lane (Figure 3, lane D). An intense signal was visible at approximately 10–13 kDa. A protein banding at 23 kDa in the gastric sample was replaced by a 20 kDa protein after duodenal digestion.

In order to deepen our knowledge about the digestibility of oleosome proteins, and specifically oleosins, upon human simulated GI digestion, selected protein bands from chewed, gastric and duodenal samples, labelled from A to M in Figure 3, were excised and in-gel digested according to the protocol described by De Angelis et al. [16] with a few modifications. Each sample (chewed, gastric and duodenal) was loaded on three different lanes of the same gel. In order to overcome some sensitivity issues and maximize the signal, each faint protein band detected in the three replicated lanes was pooled, and further submitted to in-gel digestion for protein characterization. LC-MS/MS analyses were carried out according to the experimental conditions detailed in our previous work [22]. Since different proteolytic enzymes were involved in the protocol for simulated GI digestion, an unspecific cleavage was envisaged in this case for protein band assignment. The results of the LC-MS/MS analysis are summarized in Table 2. As expected, the protein composition of the chewed sample was similar to that previously obtained for the undigested sample. Band A (ranging between 50 and 37 kDa) was mainly attributed to an almond 7S globulin and chymotrypsin, while band B (around 25 kDa) was identified as a mix of a peroxygenase, a vicilin and oleosin 1 (this last accessed with the ID A0A5E4ET55). As expected, oleosin proteins were mainly identified in band C, ranging between 15 and 10 kDa, along with the chymotrypsin enzyme, fragments of almond Prunin 1 (reported as legumin with ID accession A0A5E4FFS0), and fragments of vicilin antimicrobial peptides 2-2, also named Pru du 8 allergens. As for the gastric sample, the two protein bands above 50 kDa were analyzed but did not provide any identification by LC-MS/MS, possibly due to sensitivity issues. All the protein bands ranging between 50 and 20 kDa (band D–G) were attributed to Prunin 1 (accession ID A0A5E4FFS0) and 2 isoforms (accession ID A0A5E4FK23), highlighting that the prunin proteins likely embedded within the oleosomes (and thus not visible in the chewed protein profile) were released in the gastric compartment and gradually digested by pepsin. This is demonstrated by the increasing number of unique peptides detected for prunin 1 in spots D, E, F and G, namely 16, 21, 39 and 59, respectively (see Table 2). A similar trend was displayed for prunin 2. The presence of different bands at different MW, each attributed to prunin (isoform 1 and 2), in the gastric sample highlights that prunin was only partially digested by pepsin during the gastric phase, and that high MW fragments of the protein still persisted. This could be due to the likely protective effect exerted by the oleosomes against digestive enzymes. In band G, peptides attributed to different proteins with generic cellular functions (vicilin protein, putative disease protein, protein belonging to strubbelig-receptor family, TCM_014128 isoform, protein with ferric-oxidoreductase activity, kinesin and WAT1-related protein) along with chymotrypsin were displayed. Band H (15–10 kDa) proteins were instead attributed to Prunin 1 and an almond oleosin 1 isoform (accession ID A0A5E4EAT1). As for oleosin 1, this result suggests that this class of proteins survived the proteolytic activity of pepsin, whereas the oleosin isoform with an accession number of A0A5E4ET55 found in the chewed but not in the gastric sample was likely susceptible to pepsin. For prunin 1, only 29 unique peptides were found, pointing out that this protein was only weakly digested by pepsin. Concerning the duodenal phase, bands I and L (ranging between 30 and 20 kDa) were mainly attributed to the prunin isoforms, suggesting that for these proteins, digestion also continued in the duodenal compartment. However, they still persisted as high MW fragments, since only a few peptides were found in the region lower than 15 kDa (see band M). Along with almond prunin, other proteins belonging to vicilin, the SMG7 family, an uncharacterized protein and a DNA-directed RNA polymerase subunit beta protein were found in bands I and L. Finally, in band M (15–10 kDa), oleosin 1 (accession ID A0A5E4EAT1) proteins were identified, demonstrating that this isoform survived the duodenal enzymes. Fragments of vicilin and WAT1-related protein were also found in band M.

### 3.3. Droplet Size Distribution

**Figure 4:**
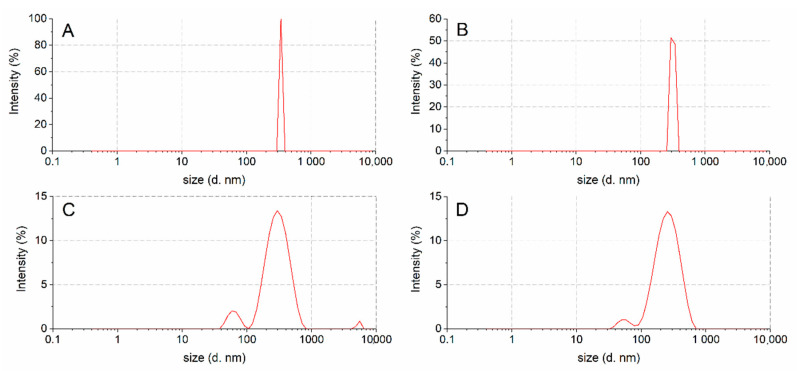
Size distribution of undigested (**A**), chewed (**B**), post in vitro gastric (**C**) and duodenal (**D**) digestion oleosomes.

Figure 4 shows the droplet size and emulsion polydispersity (PdI) of oleosomes in water obtained from undigested (Figure 4A) and digested almond oleosomes (chewed, Figure 4B, gastric, Figure 4C and duodenal, Figure 4D), taking into account both the mean hydrodynamic diameter (D_H_) and the width distribution of this complex system (Table 3). Both parameters are calculated according to the International Standard on dynamic light scattering ISO 22412 [23].

The droplet size population of untreated oleosomes showed a monomodal droplet size distribution with an average diameter of 343 nm and a polydispersity index (PdI) of 0.07 (Figure 4 and Table 3), while the chewed oleosomes showed a droplet population similar to the untreated oleosomes (mean oleosomes diameter is 318 nm with a PdI of 0.14). This behavior indicates that the salivary enzymes did not affect the original oleosome structure. These data are also supported by the SDS-PAGE protein profiles and LC-MS analyses.

The oleosomes treated with gastric enzymes showed two families of nanoassemblies with D_H_ values of 308 nm and 77 nm. In particular, a slight decrease in the droplet size with respect to the undigested sample was detected for the main population (308 vs. 318 nm, respectively). Regarding the duodenal digested samples, a decrease in the droplet size with respect to the gastric digested sample was detected (246 vs. 308nm, respectively), probably due to the loss of proteins (Figure 3, lane D), together with a secondary size distribution (intensity % of about 4) centred at 56 nm.

## 4. Discussion

The present study reports an investigation into almond oleosomes and their stability during passage through the GI tract. The imaging analysis of the lipid markers performed to visualize the almond oleosomes in the seed shows a homogeneous distribution over the whole surface of the nut, rather than a preferential distribution of the same in well-defined compartments, as is the case for other oilseeds [12]. These results are in accordance with previous observations derived from the light, scanning electron and transmission electron microscopy of almond seeds, which highlighted an even distribution of oil droplets within the natural almond cells [5,8]. In almond cells separated by the Ca-chelating agent CDTA (50mM Na_3_HCDTA 5 mM Na_2_S_2_O_5_, pH 7), the lipids were clearly located in spherical structures (mean size 2.5 µm) identifiable by their size and shape as oleosomes using bright-field or fluorescence microscopy [4]. The lipid distribution in natural almond cells subjected to gastric and duodenal digestion demonstrated the presence of some cells, mainly from the centers of the almond tissue blocks, containing oleosomes as found in the untreated raw almonds. Other cells, from the peripheral layers of the almond tissue blocks, contained large masses of coalesced lipid, a sign of lipid digestion. However, the mastication of natural almonds did not result in lipid coalescence in the majority of undamaged cells, and no significant changes were observed after gastric incubation of the samples [4,5]. Particle size seemed to be a crucial factor, since most of the enzyme activity was detected in peripheral cell layers [5]. These observations seem to agree with the droplet size distribution of the almond oleosomes reported here, although no statistically significant difference was found.

The findings on the fate of almond oleosomes during GI digestion, combined with our previous in vitro and in vivo observations, provide further evidence supporting the role played by almond cell walls in the encapsulation of intracellular lipid, therefore reducing the rate and extent of lipid release and digestion in the upper GI tract [7]. The delivery of plant material, including almond dietary fiber and undigested intracellular nutrients, to the large intestine has important implications for the gut microbiota’s composition and metabolism, increasing energy absorption and satiety [24,25].

The elucidation of the protein profile before and after in vitro digestion showed that oleosins persisted throughout chewing, with changes occurring after the gastric and duodenal phase, and a protective effect is exerted by the oleosomes against proteolytic enzymes. Interestingly, new bands attributed to almond prunin isoforms were detected after gastric digestion, suggesting that some displacement of oleosomes occurs during this phase, thus releasing these proteins, which were then partially digested by pepsin. Prunin digestion continued during the duodenal phase, as demonstrated by the corresponding bands of the duodenal electrophoretic profile, although it appeared not completely digested at the end of the process. In a previous investigation, we have reported that prunin was sensitive to pepsin in almond flour, and the addition of the surfactant phosphatidylcholine did not affect the rate or kinetics of digestion in the stomach and the small intestine [4]. Prunin is a protein able to withstand a number of food processing methods and model thermal treatments, making it a good marker to investigate almond protein digestibility [26]. In agreement with the SDS-PAGE and LC-MS analyses, the droplet size of almond oleosomes showed a monomodal distribution throughout digestion. This behavior tends to lead to homogeneous coalescence over time.

It is possible to speculate that the gastric and duodenal enzymes lead to a partial oleosins breakdown during gastric and duodenal digestion. As shown in Figure 3, new proteins were detected after gastric and duodenal digestion; in particular, high and low MW proteins were detected after gastric digestion (Figure 3, lane G), whereas the high MW proteins almost disappeared after duodenal digestion (only faint bands were detected), with the simultaneous appearance of a greater concentration of low molecular weight proteins (Figure 3, lane D).

This behavior could be explained by a rearrangement of the oleosomes protein pattern after gastric and duodenal digestion. Light scattering, SDS-PAGE and LC/MS analyses support this hypothesis, showing an apparent decrease in the droplet mean diameter from chewed to gastric and from gastric to duodenal samples, while assuming a maintenance of the basic structure of the oleosome, which could explain the high variability found in the reduction in the particle size of oleosomes, leading to no statistically significant differences.

Beisson et al. [3] reported that proteolytic enzymes are involved in the breakdown of proteins on the surface of oil bodies, and phospholipid hydrolysis occurs only when oleosins are removed. Furthermore, bile salts are likely to displace any amphiphilic molecules present at the interface, including oleosins and phospholipids, and the interface covered by the bile salts would promote lipolysis [3]. This evidence would explain the protective role played by oleosomes in the proteolytic cleavage of almond oleosins during mastication and gastric digestion, with partial digestion and rearrangement of the phospholipid profile in the small intestine. These results provide a mechanistic understanding of the impact of slow energy release and lipid encapsulation on appetite control and satiety.

## 5. Conclusions

The aim of the present study was to investigate the oleosomes distribution within natural almonds, and their fate during GI digestion, in order to establish their potential role in regulating lipid bioaccessibility.

As expected, imaging analysis confirmed an even distribution of oleosomes within the natural almond seed. The profiles of the proteins embedding the oleosomes changed after simulated gastric and duodenal digestion, whereas no differences were detected between the undigested and the chewed sample. The structure of the oleosomes remained monomodal for the chewed sample, with a slight droplet size reduction, which could be related to a change in the protein profile. On the contrary, a bimodal size distribution was observed after simulated gastric and duodenal digestion, characterized by a main population which exhibited a decrease in nanoassemblies size compared to the untreated oleosomes, and a minority population of smaller size, probably due to a partial disassembling of the structure. The rearrangement of the protein fraction embedded in the phospholipid layer during digestion could preserve the lipid fraction, thus limiting almond nutrient bioaccessibility.

This work provides further evidence of the role played by food structure during the digestion of plant foods, and in particular almond kernels. It is well-known, indeed, that particle size and the degree of processing affect the rate and extent of lipid released from almonds during digestion and fatty acid production, and that these mechanisms have a profound impact with respect to human metabolism [5].

## Figures and Tables

**Table 1:** Identification of protein bands excised from the sodium dodecylsulphate-polyacrylamide gel electrophoresis (SDS-PAGE) of oleosome proteins extracted with 2% SDS buffer (see Figure 2) and analyzed by Liquid Chromatography High-Resolution Tandem Mass Spectrometry (LC-HR-MS/MS) through detection of the prototypic peptides. All relevant parameters retrieved by software analysis are reported.

Band	Accession	Description (OS *Prunus dulcis*)	Coverage (%)	Peptides (Unique)	PSMs	MW (kDa)	Score
1	A0A5E4EAH6	PREDICTED: basic 7S globulin	58.7	21 (21)	107	46.9	70.2
2	A0A5E4EKE0	PREDICTED: peroxygenase	15.9	3 (2)	13	26.5	5.9
	A0A4Y1QWY8	Beta-tonoplast intrinsic protein	8.9	2 (2)	8	27.2	2.7
3	A0A5E4FFS0	PREDICTED: legumin	8.5	4 (4)	14	63	5.6
	A0A4Y1QWY8	Beta-tonoplast intrinsic protein	23.4	4 (4)	15	27.2	4.4
4	A0A5E4ET55	Oleosin	50.6	5 (4)	27	16.6	9.5
	A0A5E4FFS0	PREDICTED: legumin	5.6	2 (2)	4	63	1.3
	A0A5E4EAT1	Oleosin	52.0	4 (4)	16	15.6	4.1

**Table 2:** Identification of selected protein bands from the SDS-PAGE of oleosome proteins extracted from almonds after simulated digestion (see Figure 3). Analyses were performed by LC-MS/MS coupled with software-based protein identification. All relevant parameters were summarized.

Digestion Step	Band	Accession	Description (OS = *Prunus dulcis*)	Coverage (%)	Peptides (Unique)	PSMs	MW (kDa)	Score
**Chew**	**A**	A0A5E4EAH6	PREDICTED: basic 7S globulin. OS = *Prunus dulcis*.	77.0	43 (42)	230	46.9	88.8
		P00766	Chymotrypsinogen A	30.6	2 (1)	20	25.7	5.2
	**B**	A0A5E4EKE0	PREDICTED: peroxygenase OS = *Prunus dulcis*.	59.0	16 (13)	75	26.5	36.5
		A0A5E4ET55	Oleosin OS = Prunus dulcis	23.7	2 (2)	6	16.6	1.3
		A0A5E4FV72	PREDICTED: vicilin OS = *Prunus dulcis*.	6.4	2 (2)	8	60	9.7
	**C**	A0A5E4ET55	Oleosin OS = *Prunus dulcis*	57.0	5 (4)	27	16.6	18.0
		A0A5E4EAT1	Oleosin OS = *Prunus dulcis*	43.2	4 (4)	22	15.6	6.3
		P00766	Chymotrypsinogen A	29.0	5 (3)	16	25.7	10.6
		A0A5E4EYX0	PREDICTED: vicilin antimicrobial peptides 2-2 OS = *Prunus dulcis*.	19.7	5 (5)	19	31.1	7.9
		A0A5E4FFS0	PREDICTED: legumin OS = *Prunus dulcis*.	19.2	3 (3)	17	63	1.4
**Gastric**	**D**	A0A5E4FFS0	PREDICTED: legumin OS = *Prunus dulcis*.	40.3	16 (16)	75	63	24.5
		P00766	Chymotrypsinogen A OS = Bos Taurus	24.5	2 (2)	6	25.7	3.7
	**E**	A0A5E4FFS0	PREDICTED: legumin OS = *Prunus dulcis*.	54.4	21 (21)	144	63	76.6
	**F**	A0A5E4FFS0	PREDICTED: legumin OS = *Prunus dulcis*.	68.6	43 (39)	272	63	156.8
		A0A5E4FK23	PREDICTED: legumin OS = *Prunus dulcis*.	25.5	4 (2)	25	35.9	7.8
	**G**	A0A5E4FFS0	PREDICTED: legumin OS = *Prunus dulcis*.	76.4	63 (59)	408	63	272.5
		A0A5E4FK23	PREDICTED: legumin OS = *Prunus dulcis*.	44.5	8 (4)	84	35.9	36.2
		A0A5E4FV72	PREDICTED: vicilin OS = *Prunus dulcis*.	18.5	3 (3)	19	60	5.7
		A0A5E4F5V3	PREDICTED: putative disease OS = *Prunus dulcis*.	2.7	2 (1)	17	128.4	2.6
		A0A5E4G4H3	PREDICTED: STRUBBELIG-RECEPTOR FAMILY OS = *Prunus dulcis*.	4.1	2 (1)	6	80.4	1.3
		A0A5E4G2W8	PREDICTED: TCM_014128 isoform OS = *Prunus dulcis*	1.0	2 (2)	5	119.9	3.1
		A0A5E4FDL3	PREDICTED: ferric OS = *Prunus dulcis*.	4.3	2 (1)	10	74	1.5
		A0A5E4ETP4	PREDICTED: kinesin OS = *Prunus dulcis*.	2.4	2 (1)	9	158.6	1.3
		P00766	Chymotrypsinogen A OS = *Bos Taurus*	26.9	4 (4)	13	25.7	3.7
		A0A5E4ERF0	WAT1-related protein OS = *Prunus dulcis*.	5.0	2 (1)	6	39.3	1.3
	**H**	A0A5E4FFS0	PREDICTED: legumin OS = *Prunus dulcis*.	60.0	29 (29)	180	63	75.9
		A0A5E4EAT1	Oleosin OS = Prunus dulcis	44.6	3 (3)	11	15.6	7.4
**Duodenal**	**I**	A0A5E4FFS0	PREDICTED: legumin OS = *Prunus dulcis*.	47.6	58 (56)	366	63	268.1
		A0A5E4FK23	PREDICTED: legumin OS = *Prunus dulcis*.	41.6	13 (12)	88	35.9	44.5
		A0A5E4FDR1	PREDICTED: legumin OS = *Prunus dulcis*.	22.4	3 (2)	25	20.5	9.9
		A0A5E4F2T7	PREDICTED: vicilin OS = *Prunus dulcis*.	14.8	3 (3)	16	62.1	6.5
		A0A5E4FH19	PREDICTED: SMG7 OS = *Prunus dulcis*.	4.0	2 (1)	18	126.2	2.9
		A0A5E4GE42	Uncharacterized protein OS = *Prunus dulcis*	54.7	15 (12)	74	34	47.7
		P00766	Chymotrypsinogen A OS = Bos Taurus	31.0	5 (2)	17	25.7	8.5
	**L**	A0A5E4FFS0	PREDICTED: legumin OS = *Prunus dulcis*.	37.0	50 (47)	318	63	253.7
		A0A5E4FK23	PREDICTED: legumin OS = *Prunus dulcis*.	36.6	9 (7)	59	35.9	32.5
		A0A5E4FV72	PREDICTED: vicilin OS = *Prunus dulcis*.	27.5	4 (4)	30	60	4.7
		A0A5E4EZP4	PREDICTED: vicilin OS = *Prunus dulcis*.	11.3	3 (3)	15	93.7	3.2
		A0A5E4F3E3	DNA-directed RNA polymerase subunit beta.	2.3	2 (1)	3	129.8	2.1
	**M**	P00766	Chymotrypsinogen A OS = *Bos taurus*	60.8	13 (8)	51	25.7	34.9
		A0A5E4FFS0	PREDICTED: legumin OS = *Prunus dulcis*.	48.8	22 (18)	154	63	74.8
		A0A5E4EAT1	Oleosin OS = *Prunus dulcis*	35.1	5 (4)	21	15.6	8.4
		P17538	Chymotrypsinogen B OS = *Homo sapiens*	25.8	5 (1)	27	27.7	17.2
		A0A5E4FK23	PREDICTED: legumin OS = *Prunus dulcis*.	19.9	2 (2)	24	35.9	5.3
		A0A5E4FDR1	PREDICTED: legumin OS = *Prunus dulcis*.	14.7	3 (1)	15	20.5	4.9
		A0A5E4EU98	WAT1-related protein OS = *Prunus dulcis*.	3.0	2 (1)	4	40.7	3.2
		A0A5E4EZP4	PREDICTED: vicilin OS = *Prunus dulcis*.	8.1	3 (1)	14	93.7	1.3

**Table 3:** The effect of chewing, gastric and gastric plus duodenal-simulated digestion on the droplet size (mean hydrodynamic diameter) and emulsion polydispersity (polydispersity index) of almond oleosomes ^a^.

Sample	D_H_ (nm ± SD) (%) ^b^	Polydispersity Index
Untreated	343 ± 12 (100%)	0.07
Chewed	318 ± 23 (100%)	0.14
Gastric	308 ± 57 (91%) 77 ± 19 (9%)	0.37 ≤0.4
Duodenal	246 ± 47 (96%) 56 ± 11 (4%)	0.38 0.39

^a^ The results are reported as the mean of three independent measurements on three different batches ± the standard deviation (SD). ^b^ Size with corresponding intensity % distribution.
